# Safety and Changes in Health-Related Quality of Life After Epidural Oxygen Injection for Long COVID/Post-COVID Syndrome: A Multicenter Retrospective Study

**DOI:** 10.3390/jcm15187326

**Published:** 2026-09-21

**Authors:** Tasuku Inaba, Kiyoshi Takagi, Masamichi Atsuchi, Shoko Atsuchi, Noriko Tamura, Koichi Takahashi, Kentaro Yoshida, Masahiro Abo

**Affiliations:** 1 Department of Rehabilitation Medicine, The Jikei University School of Medicine, Tokyo 105-8461, Japan; y.jreha@gmail.com (K.Y.); abo19masa5@gmail.com (M.A.); 2 Department of Neurosurgery, Atsuchi Katsushika Clinic, Tokyo 124-0006, Japan; paulktkg@mac.com (K.T.); atsuchi0824@yahoo.co.jp (M.A.); atsuchicd6@yahoo.co.jp (S.A.); ztb05666@gmail.com (N.T.); koichi@takahashik.com (K.T.); 3 Department of Neurosurgery, Atsuchi Neurosurgical Hospital, Kagoshima 892-0842, Japan; 4 Normal Pressure Hydrocephalus Center, Abiko Seijinkai Hospital, Chiba 270-1177, Japan; 5 Department of Neurosurgery, Sanno Hospital, Tokyo 107-0052, Japan

**Keywords:** long-COVID, post-COVID-19 syndrome, autonomic dysfunction, epidural oxygen injection

## Abstract

**Background/Objectives**: Long COVID/post-COVID-19 syndrome is associated with persistent symptoms, including fatigue, headache, and autonomic dysfunction, leading to impaired health-related quality of life (HRQoL). Although epidural oxygen injections have been used in patients with chronic post-traumatic headache and mild traumatic brain injury, their safety and therapeutic effects have not been systematically evaluated. **Methods**: In this multicenter retrospective study, patients with Long COVID/post-COVID-19 syndrome who underwent their first epidural oxygen injection between April 2022 and December 2025 were included. Safety was assessed by reviewing adverse events. HRQoL was evaluated using the Japanese version of the 36-Item Short-Form Health Survey version 2 (SF-36v2^®^) before treatment and approximately one month afterward. Paired SF-36v2^®^ scores were compared using the Wilcoxon signed-rank test with Bonferroni correction. **Results**: Forty-two patients were included (mean age, 31.9 ± 16.2 years; 21 males). The mean interval between COVID-19 infection and treatment was 16.7 ± 12.8 months. Adverse events occurred in eight patients (subcutaneous emphysema, *n* = 5; dyspnea, *n* = 2; back pain, *n* = 1); they all resolved spontaneously within one week, with no deaths, hospitalizations, or long-term sequelae. Paired SF-36v2^®^ data were available for 35 patients. The median scores improved across all domains, with the greatest increase observed in vitality (+25.0 points). All improvements remained significant after Bonferroni correction. **Conclusions**: Epidural oxygen injection demonstrated a favorable short-term safety profile and was associated with improved HRQoL in patients with Long COVID/post-COVID-19 syndrome. Prospective controlled studies using objective outcome measures are needed to confirm efficacy and clarify the underlying mechanisms.

## 1. Introduction

Long COVID/post-COVID-19 syndrome is characterized by a wide range of persistent symptoms following acute SARS-CoV-2 infection, including fatigue, headache, tinnitus, respiratory symptoms, and cardiovascular symptoms [1,2,3,4,5]. In addition, post-exertional malaise, defined as the exacerbation of symptoms and profound fatigue following physical or mental exertion, has been increasingly recognized as a characteristic feature of the condition [6,7]. The pathophysiology of Long COVID/post-COVID-19 syndrome is thought to be multifactorial, involving mechanisms such as neuroinflammation, autoimmunity, autonomic dysfunction, and microvascular thrombosis [8,9,10]. Owing to its substantial impact on health-related quality of life (HRQoL) [11,12,13], Long COVID/post-COVID-19 syndrome has emerged as a significant public health concern, highlighting the need for effective therapeutic strategies to alleviate symptoms and facilitate social reintegration.

A clinically important subset of Long COVID/post-COVID-19 syndrome presents with symptoms that overlap with chronic post-traumatic headache (CPTH), postconcussion syndrome and mild traumatic brain injury (mTBI). Symptoms include headache, fatigue, dizziness, visual discomfort, impaired concentration, exercise intolerance, and autonomic complaints [14,15,16,17]. CPTH and mTBI have traditionally been interpreted as consequences of structural or functional brain injury [16,18]. However, previous studies have suggested that persistent symptoms after minor craniocervical trauma may not always be explained by the brain injury itself or by classical spontaneous intracranial hypotension [14,19]. Instead, abnormalities in cerebrospinal fluid (CSF) dynamics and autonomic function may contribute to symptom persistence in selected patients, even in the absence of typical findings of spontaneous intracranial hypotension [14,15,16,17,18,19]. Orthostatic intolerance and autonomic dysfunction may provide an additional clinical bridge between Long COVID/post-COVID-19 syndrome and postconcussion syndromes. Postural tachycardia syndrome has been reported after mTBI in adolescents with persistent post-concussion symptoms [20,21]. Long COVID/post-COVID-19 syndrome has also been associated with orthostatic intolerance, postural tachycardia syndrome, and dysautonomia [3,4]. These observations suggest that Long COVID and CPTH/mTBI-like syndromes share an orthostatic-autonomic phenotype in a subset of patients.

Gas-based neuraxial interventions have a long but largely forgotten history. Penfield reported lumbar air insufflation encephalography as a specific treatment for chronic meningeal post-traumatic headache in 1927 [22]. Matyas reported epidural air injection through the sacral hiatus as a treatment for low back pain and sciatica in 1953 [23]. These historical and clinical observations suggest that gas-based neuraxial interventions may modify symptoms in selected patients, although their mechanisms remain uncertain. It has been reported that epidural saline and oxygen injections were administered to patients with CPTH/mTBI-like symptoms, whose symptoms resembled spontaneous intracranial hypotension, although their intracranial pressure was not necessarily low and their brain magnetic resonance imaging (MRI) findings could be negative [15,19]. Historical reports also described a transition from room air to oxygen for neuraxial gas procedures because oxygen was empirically associated with less post-procedural discomfort, although the underlying mechanism was not investigated [24]. The present procedure should be distinguished from oxygen–ozone (O_2_–O_3_) therapy. O_2_–O_3_ therapy has been investigated as a minimally invasive treatment for lumbar disc herniation and radicular low back pain and involves the administration of a gas mixture containing ozone [25,26]. Its proposed mechanisms are primarily related to the biochemical and oxidative properties of ozone [25]. In contrast, the procedure evaluated in this study used 100% medical oxygen without ozone. The selection of oxygen was based on its historical use in neuraxial gas procedures, including therapeutic oxygen encephalography, along with our previous clinical experience with epidural saline and oxygen injections [15,24]. Thus, the present intervention is termed “epidural oxygen injection” to clearly distinguish it from oxygen–ozone therapy.

Based on these historical and clinical observations, epidural oxygen injection was offered as an exploratory therapeutic option to patients with Long COVID/post-COVID-19 syndrome whose predominant symptoms overlapped with CPTH/mTBI-like and orthostatic-autonomic phenotypes and who elected to undergo the procedure after receiving an explanation of its exploratory nature. Therefore, we conducted a multicenter retrospective exploratory study to evaluate the short-term safety of epidural oxygen injection and its association with changes in HRQoL among these patients.

## 2. Materials and Methods

This was a multicenter retrospective study. Eligible patients met the following inclusion criteria: (1) underwent their first epidural oxygen injection at Atsuchi Neurosurgical Hospital or Atsuchi Katsushika Clinic between 1 April 2022, and 31 December 2025, and (2) had been diagnosed with Long COVID/post-COVID-19 syndrome at our institution or at another medical institution. The exclusion criterion was age ≤ 6 years at the time of treatment.

Data collected from the electronic medical records included demographic characteristics, the interval between COVID-19 infection and epidural oxygen injection treatment, symptoms prior to treatment, the puncture site and volume of oxygen administered during treatment, treatment-related adverse events, and responses to the Japanese version of the 36-Item Short-Form Health Survey version 2 (SF-36v2^®^) questionnaire obtained before and after treatment [27,28].

### 2.1. Diagnosis

**Figure 1:**
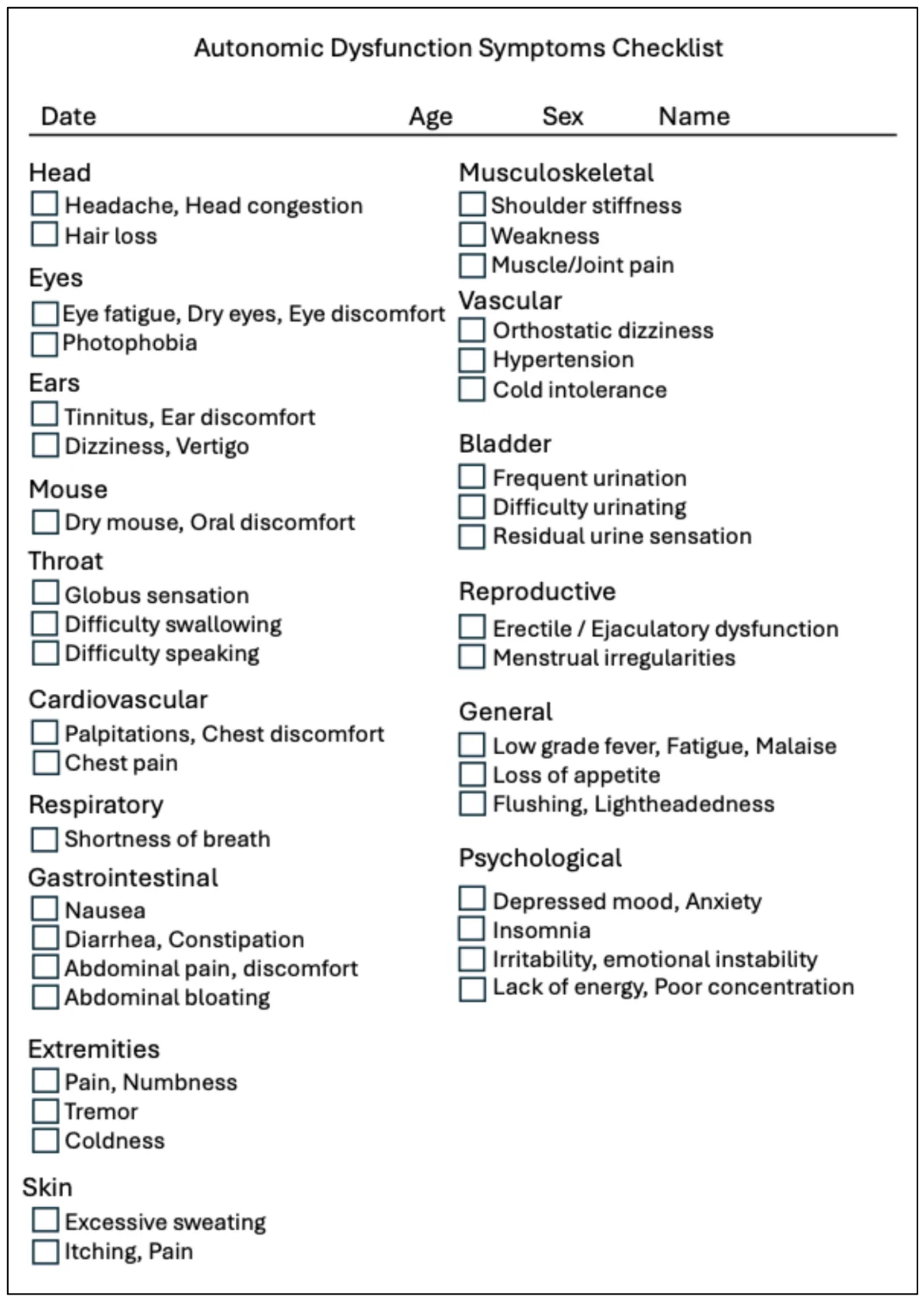
Autonomic symptoms checklist.

Patients who had been diagnosed with Long COVID/post-COVID-19 syndrome at our institution or another medical institution were eligible for inclusion. The diagnosis was based on the World Health Organization (WHO) definition, namely the presence of symptoms that developed within 3 months after the onset of COVID-19 and persisted for at least 2 months [29]. Before treatment, all patients underwent blood tests, brain magnetic resonance imaging (MRI), and whole-spine MRI to exclude other organic diseases. Additionally, the MRI findings were reviewed to confirm the absence of CSF leakage and intradural or extradural space-occupying lesions, thereby ensuring the safety of the procedure. Pretreatment autonomic symptoms were evaluated using a structured symptom checklist covering multiple domains, including neurological, cardiovascular, gastrointestinal, and psychological symptoms (Figure 1).

### 2.2. Epidural Oxygen Injection Procedure

**Figure 2:**
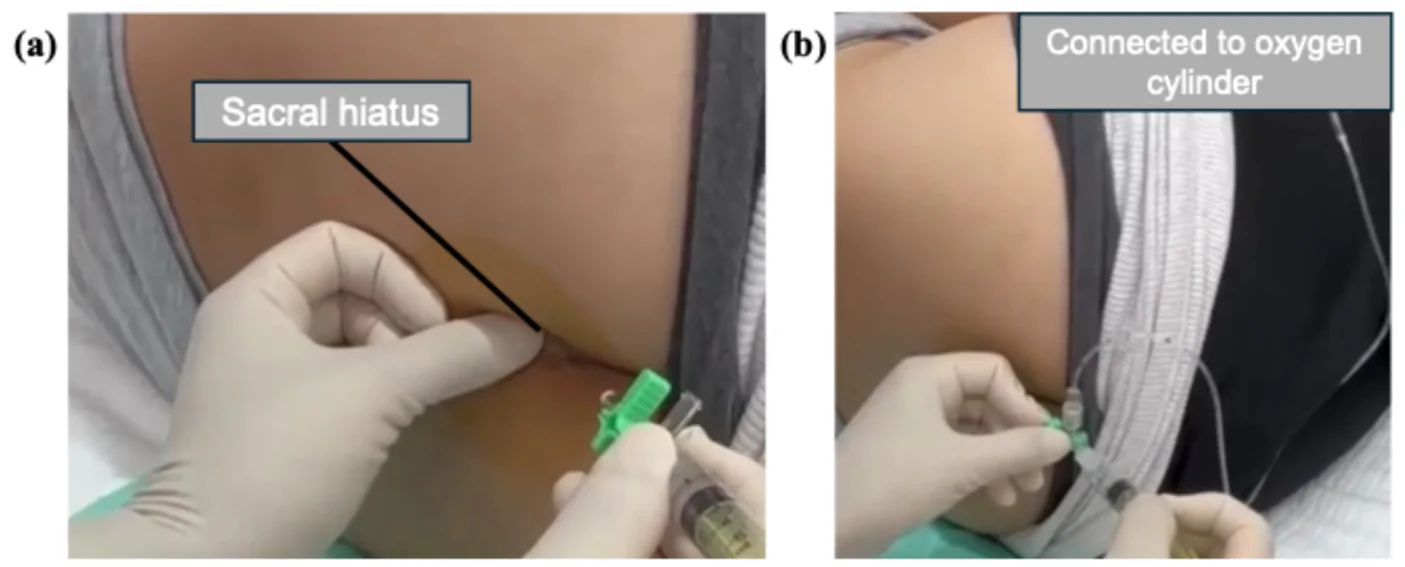
Epidural oxygen injection procedure (sacral hiatus puncture). (**a**) After local anesthesia, a spinal needle was inserted into either the lumbar interlaminar epidural space or the sacral hiatus. (**b**) After confirmation of epidural placement using the loss-of-resistance technique, 100% oxygen was administered in 5-mL increments; patient tolerance or resistance to injection determined the final injection volume.

The epidural oxygen injection procedure involved puncturing either the interlaminar lumbar epidural space or the sacral hiatus, followed by the administration of 100% oxygen (Figure 2). The detailed procedure was as follows.

After local anesthesia was administered with 1% lidocaine at the puncture site, a 21-gauge spinal needle was inserted into either the lumbar interlaminar space or the sacral hiatus (Figure 2a). For the lumbar puncture, the epidural space was identified using the loss-of-resistance technique with normal saline. After confirmation of epidural placement, 100% oxygen was administered through the needle in 5-mL increments using a syringe (Figure 2b). The volume of oxygen administered was determined individually based on patient tolerance. The oxygen injection was continued until the patient reported discomfort or resistance to the injection. All procedures were performed on an outpatient basis. Following treatment, the patients remained at rest for approximately 40 min and were monitored for adverse events before discharge.

### 2.3. Data Collection Using Short Form-36

For the pre-treatment assessment, patients completed the SF-36v2^®^ questionnaire either on the day of epidural oxygen injection treatment or on the preceding day. For the post-treatment assessment, patients were scheduled for an outpatient follow-up visit approximately 1 month after the procedure and were asked to complete the SF-36v2^®^ questionnaire at that time. The approximately 1-month follow-up interval was selected to evaluate the initial clinical response and short-term safety of this exploratory intervention while minimizing the influence of variations in follow-up timing. For patients who were unable to attend the follow-up visit or resided far from the study institutions, the questionnaire was completed and returned by mail.

### 2.4. Outcome Assessment

The primary outcomes were the numbers and types of adverse events occurring during the procedure or throughout the post-treatment observation period as measures of safety and the change in HRQoL following epidural oxygen injection treatment; this was assessed using the SF-36v2^®^ questionnaire. Analyses were conducted on patients with available SF-36v2^®^ data at both baseline and follow-up. Changes in each SF-36v2^®^ domain score were calculated by comparing pre-treatment and post-treatment assessments. The secondary outcome was the autonomic symptoms reported at baseline.

### 2.5. Data Analysis

Statistical analyses were performed using R (version 4.5.3; R Foundation for Statistical Computing, Vienna, Austria). Continuous variables were summarized as the mean ± standard deviation (SD) or median with interquartile range (IQR), as appropriate. Changes in SF-36v2^®^ scores before and after epidural oxygen injection treatment were analyzed using the nonparametric Wilcoxon signed-rank test for paired samples. Effect sizes were calculated as r using the standardized test statistic divided by the square root of the number of paired observations, with values of approximately 0.1, 0.3, and 0.5 interpreted as small, medium, and large effects, respectively. Statistical significance was set at *p* < 0.05. To account for multiple comparisons across the SF-36v2^®^ domains, Bonferroni-adjusted *p* values were used.

### 2.6. Ethical Considerations

This study was conducted in accordance with the principles of the Declaration of Helsinki and was approved by the Ethics Committee of Jikei University School of Medicine (approval No. 37-328 [12973]).

## 3. Results

### 3.1. Patient Characteristics

A total of 42 patients (21 men and 21 women) were included in the study. The mean patient age at the time of treatment was 31.9 ± 16.2 years. The mean interval between COVID-19 infection and epidural oxygen injection treatment was 16.7 ± 12.8 months (Table 1). Responses to the autonomic symptom checklist were available for 33 of the 42 patients. The most frequently reported symptoms were “headaches, head congestion” (28 patients, 67%), “low-grade fever, fatigue, malaise” (25 patients, 60%), and “shoulder stiffness” (21 patients, 50%) (Table 2). Pre- and post-treatment SF-36v2^®^ data were available for 35 patients. Among the seven patients excluded from the HRQoL analysis, four did not complete the pretreatment SF-36v2^®^ questionnaire, and post-treatment SF-36v2^®^ data were unavailable for the remaining three patients.

### 3.2. Safety Assessment of Epidural Oxygen Injection

Among the 42 patients who underwent epidural oxygen injection, the sacral hiatus was used as the puncture site in 39 patients, whereas lumbar epidural puncture was performed in 3 patients (Table 3). The volume of oxygen administered ranged from 40 to 80 mL, with 60 and 70 mL being the most frequently administered volumes. Adverse events were observed in eight patients. Subcutaneous emphysema occurred in 5 patients, dyspnea in 2, and back pain in 1. All adverse events resolved spontaneously within 1 week. No deaths, long-term sequelae, or adverse events requiring hospitalization were observed.

### 3.3. HRQoL Outcomes Following Epidural Oxygen Injection

**Figure 3:**
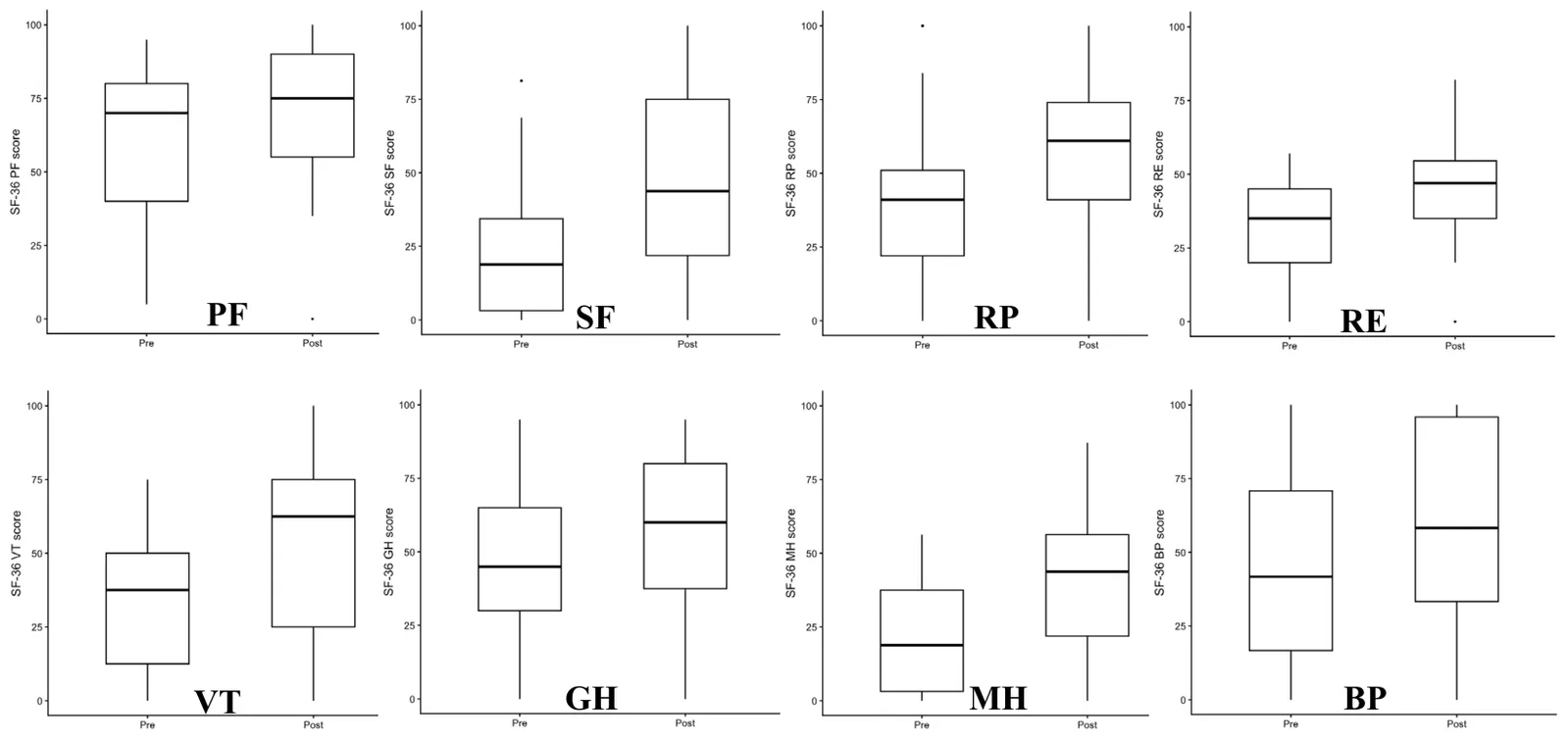
Box plots of changes in each SF-36v2^®^ domain. Changes in SF-36v2^®^ domain scores befor e and after epidural oxygen injection. Box-and-whisker plots show the median, interquartile range, and range of each SF-36v2^®^ domain before treatment and approximately 1 month after treatment. Median scores increased across all eight domains, and all paired comparisons remained statistically significant after Bonferroni correction.

Post-treatment SF-36v2^®^ data were collected approximately 30 days after epidural oxygen injection treatment. Among the 35 patients with paired pre- and post-treatment SF-36v2^®^ data, median scores increased after treatment in all SF-36v2^®^ domains (Table 4, Figure 3). The median changes in domain scores were as follows: +5.0 points for physical functioning; +18.8 points for social functioning; +12.0 points for physical role limitation; +15.0 points for emotional role limitation; +25.0 points for vitality; +10.0 points for general health; +18.8 points for mental health; and +8.3 points for bodily pain. The greatest median improvement was observed in the vitality domain. The Wilcoxon signed-rank test demonstrated significant improvements in all SF-36v2^®^ domains, which remained statistically significant after Bonferroni correction for multiple comparisons.

## 4. Discussion

In this multicenter retrospective study, we evaluated the safety and potential therapeutic effects of epidural oxygen injection in patients with Long COVID/post-COVID-19 syndrome. Safety was assessed by reviewing the incidence and clinical course of adverse events after the procedure, whereas the impact of the treatment on HRQoL was evaluated by comparing SF-36v2^®^ scores before and approximately 1 month after epidural oxygen injection. These results suggest that epidural oxygen injection may be a feasible and relatively safe exploratory intervention and may be associated with short-term improvements in HRQoL among selected patients with Long COVID/post-COVID-19 syndrome.

The adverse events observed after epidural oxygen injection in our study were limited to transient and minor events. Although the observation period after treatment was relatively short, with a minimum follow-up of approximately 1 month, no patient experienced long-term sequelae or required hospitalization. However, because the present cohort included only 42 patients, it was not sufficiently powered to evaluate rare but serious complications. Procedures involving puncture of and injection of material into the epidural space include lumbar epidural anesthesia, caudal epidural block, and epidural blood patch. In lumbar epidural anesthesia and other central neuraxial blocks, reported complications include epidural hematoma, epidural abscess, meningitis, nerve injury, spinal cord ischemia, postdural puncture headache, and systemic toxicity of the local anesthetic [30,31]. With regard to caudal epidural block, local anesthetic systemic toxicity, cauda equina syndrome, and transient neurological symptoms have been reported [32]. Following an epidural blood patch, back pain, radicular symptoms, rebound intracranial hypertension, infection, meningitis, spinal or subdural hematoma, arachnoiditis, cauda equina syndrome, and paraplegia have been reported, particularly in the context of large-volume epidural blood patching [33,34]. Therefore, when administering epidural oxygen injections, physicians should be aware of the complications that may occur with related epidural procedures. In addition, because this procedure involves gas injection, gas-related adverse events such as subcutaneous emphysema may occur. In our study, all cases of subcutaneous emphysema resolved spontaneously, and no serious complications were observed. Additionally, transient dyspnea was observed in two patients in the present study. Matyas reported dyspnea in 2 of 132 patients undergoing epidural air injection. One patient experienced dyspnea and vomiting for approximately 2 min, whereas another developed dyspnea with a severe sensation of suffocation lasting approximately 1 h after the injection of 50 mL of air. Matyas considered air embolism to be the cause of these events, although no confirmatory diagnostic evidence was presented. He therefore emphasized the importance of avoiding intravascular injection and recommended monitoring patients’ pulse and respiration for 1–2 min between injections [23]. In our study, no serious cardiopulmonary or neurological complications were observed; however, the mechanism underlying the transient dyspnea could not be determined. Potential mechanisms include inadvertent intravascular gas injection, upward migration of the gas causing mediastinal emphysema or cervical compression, or a vasovagal reflex. Therefore, intravascular gas injection and other gas-related complications should be considered whenever respiratory symptoms occur during or after the procedure. Furthermore, Takagi et al. reported no serious procedure-related complications in 23 patients treated with epidural oxygen injections [15]. Collectively, these historical observations are consistent with the present findings and support the favorable short-term safety profile of gas-based epidural interventions. Nevertheless, cumulative clinical experience remains limited, and larger prospective studies with systematic safety monitoring are required to define the incidence of rare neurological and pressure-related complications more precisely. An additional practical feature of the present procedure is that it can be performed under local anesthesia without general anesthesia or airway instrumentation. This may be clinically relevant in patients with Long COVID/post-COVID-19 syndrome, particularly those with persistent respiratory or cardiopulmonary symptoms. Previous large international surgical cohorts have demonstrated the substantial clinical impact of pulmonary complications and perioperative SARS-CoV-2 infection on postoperative mortality [35,36]. Although these findings were derived from surgical populations and cannot be directly extrapolated to the present outpatient procedure, avoiding general anesthesia may represent a practical advantage when considering procedural burden and feasibility in this population. Taken together, epidural oxygen injections did not demonstrate serious safety signals during the short-term follow-up period in the present cohort, although rare adverse events cannot be excluded.

Long COVID/post-COVID-19 syndrome is characterized by a broad spectrum of autonomic symptoms that vary substantially among individuals [1,2,3,4,5,37]. Furthermore, symptom severity may fluctuate over time in the same individual, resulting in considerable inter- and intra-individual variability [38,39,40]. In addition, previous studies have suggested that objective measures of autonomic dysfunction, such as heart rate variability [41], do not necessarily correlate with subjective symptom burden in patients with Long COVID/post-COVID-19 syndrome [42,43]. Consequently, no consensus has been reached regarding objective biomarkers that reliably reflect clinical improvement or deterioration in this population. Given these challenges, we selected the SF-36v2^®^ as a comprehensive patient-reported outcome measure to evaluate overall changes in HRQoL within individual patients. In our study, improvements were observed across all SF-36v2^®^ domains following epidural oxygen injection, suggesting that this treatment may have the potential to improve HRQoL in patients with Long COVID/post-COVID-19 syndrome. However, because SF-36v2^®^ is a comprehensive measure of HRQoL rather than a symptom-specific assessment, further studies incorporating control groups and disease-specific outcome measures are necessary to better characterize the therapeutic effects of this intervention. As a preliminary step toward this objective, we employed a structured autonomic symptom checklist to comprehensively assess symptom profiles in the present study. The most commonly reported symptoms included headaches, fatigue, malaise, and shoulder stiffness. In future investigations, we plan to incorporate symptom-specific outcome measures for these manifestations by drawing on established assessment tools used in migraine and myalgic encephalomyelitis/chronic fatigue syndrome research [44,45,46].

This study has two notable novel findings. The first novel aspect of this study is that, to our knowledge, it provides the first systematic clinical evaluation of epidural oxygen injections. Although gas-based neuraxial interventions have a long but largely forgotten history, including lumbar air insufflation described by Penfield and epidural air injection reported by Matyas, these procedures have never been systematically evaluated using standardized patient-reported outcomes [22,23]. Building on the clinical experience reported in patients with CPTH/mTBI-like syndromes [14], our study extends these historical observations by providing preliminary data on the safety and short-term clinical outcomes of epidural oxygen injection. Proposed mechanisms include transient modulation of epidural pressure, alteration of CSF dynamics, and epidural pressure-mediated mechanical neuromodulation of nerve root and autonomic function [47,48,49]. However, these mechanisms remain speculative, and the present clinical study was not designed to investigate them directly. Therefore, the observed improvements in HRQoL should not be interpreted as evidence of any specific biological mechanism. Future mechanistic studies incorporating physiological assessments, neuroimaging, and objective autonomic biomarkers are required to clarify whether these proposed mechanisms contribute to the clinical effects of epidural oxygen injection.

The second novel aspect of our study is that it introduces epidural oxygen injection as another exploratory therapeutic strategy for Long COVID/post-COVID-19 syndrome. As no disease-modifying therapy has yet been established, several exploratory interventions have recently been investigated. Among these, epipharyngeal abrasive therapy has shown promising improvements in multiple symptoms and HRQoL in selected patients [50,51]. Similarly, our study suggests that epidural oxygen injections may represent another exploratory therapeutic strategy capable of improving multiple patient-reported domains in selected patients. As the therapeutic targets and proposed mechanisms differ substantially, direct comparisons cannot be made. Rather, these emerging interventions may represent complementary therapeutic concepts for different clinical phenotypes within the heterogeneous spectrum of Long COVID/post-COVID-19 syndrome, with epidural oxygen injection potentially being particularly relevant for patients exhibiting CPTH/mTBI-like and orthostatic-autonomic features.

This study has some limitations. First, this was a retrospective study without a control group, and treatment outcomes were evaluated primarily using subjective patient-reported measures. The absence of a control group, such as patients who underwent sham procedures, prevents precise differentiation between the true biological effect of epidural oxygen injection and a placebo response or expectation bias. In addition, epidural oxygen injection is an invasive procedure that may induce substantial expectancy and placebo effects, potentially resulting in improvements in subjective outcomes independent of any physiological effects of the treatment. Therefore, the observed improvements in SF-36v2^®^ scores should be interpreted cautiously and cannot be regarded as definitive evidence of treatment efficacy. Nevertheless, the present findings provide preliminary data regarding the safety and potential therapeutic effects of epidural oxygen injections. Building on these preliminary findings, future studies should employ prospective randomized controlled trials incorporating sham or appropriate control interventions whenever feasible. Furthermore, objective physiological biomarkers of autonomic function, such as heart rate variability, together with symptom-specific outcome measures, should be incorporated to distinguish true biological treatment effects from placebo responses and to more rigorously evaluate the efficacy and mechanisms of epidural oxygen injections.

Another limitation is that CSF pressure was not measured in the present study. The assessment of CSF pressure may be important for elucidating the mechanisms underlying the observed clinical effects. However, because the procedure involves the intentional injection of oxygen into the epidural space, lumbar puncture is not routinely performed to minimize the risk of complications such as pneumocephalus. Consequently, the present study could not determine whether abnormalities in CSF pressure were present in the study population or whether changes in CSF dynamics contributed to the treatment response. Future observational studies specifically designed to evaluate CSF pressure and related physiological parameters will be necessary to further investigate the potential role of CSF-related mechanisms in Long COVID/post-COVID-19 syndrome.

## 5. Conclusions

In this multicenter retrospective study, no serious adverse events were observed during short-term follow-up after epidural oxygen injection, and patient-reported HRQoL scores improved across all SF-36v2^®^ domains. However, as this was an uncontrolled retrospective study that relied primarily on subjective outcomes, these observations cannot establish therapeutic efficacy. Therefore, these findings should be regarded as preliminary and hypothesis-generating. Prospective controlled studies incorporating symptom-specific assessments, objective biomarkers, physiological measurements, and appropriate control or sham interventions are required to determine the efficacy and safety of epidural oxygen injections and to elucidate the mechanisms underlying the observed changes.

## Figures and Tables

**Table 1:** Characteristics of patients who underwent an epidural oxygen injection.

Characteristics	Value
Overall, *n* (female)	42 (21)
Age, Mean (SD)	31.9 (16.2)
Months between COVID-19 infection and treatment, Mean (SD)	16.7 (12.8)
Days between pre-SF36 and post-SF-36, Median (IQR)	32 (28–36.5)
**Baseline SF-36**	
Physical functioning (PF), Median (IQR)	70.0 (40.0–80.0)
Social functioning (SF), Median (IQR)	18.8 (3.1–34.4)
Physical role limitation (RP), Median (IQR)	41.0 (22.0–51.0)
Emotional role limitation (RE), Median (IQR)	35.0 (20.0–45.0)
Vitality (VT), Median (IQR)	37.5 (12.5–50.0)
General health (GH), Median (IQR)	45.0 (30.0–65.0)
Mental health (MH), Median (IQR)	18.8 (3.1–37.5)
Body pain (BP), Median (IQR)	41.7 (16.7–70.8)

**Table 2:** Results from autonomic symptoms checklist. This figure shows the pre-treatment answers to the autonomic symptom checklist. Responses were available for 33 patients. Symptoms are listed in descending order of frequency. Headache/head congestion and fatigue/malaise were the most commonly reported symptom categories at baseline.

Symptoms	*n* (Percentage)
Headache, Head congestion	28 (67%)
Low grade fever, Fatigue, Malaise	25 (60%)
Shoulder stiffness	21 (50%)
Orthostatic dizziness	20 (48%)
Lack of energy, Poor concentration	20 (48%)
Eye fatigue, Dry eyes, Eye discomfort	16 (38%)
Depressed mood, Anxiety	16 (38%)
Dizziness, Vertigo	15 (36%)
Insomnia	15 (36%)
Muscle/Joint pain	14 (33%)
Palpitations, Chest discomfort	13 (31%)
Irritability, emotional instability	13 (31%)
Weakness	12 (29%)
Abdominal pain, discomfort	11 (26%)
Photophobia	10 (24%)
Shortness of breath	10 (24%)
Diarrhea, Constipation	10 (24%)
Loss of appetite	10 (24%)
Flushing, Lightheadedness	10 (24%)
Cold intolerance	9 (21%)
Abdominal bloating	8 (19%)
Coldness	8 (19%)
Tinnitus, Ear discomfort	7 (17%)
Hair loss	6 (14%)
Nausea	6 (14%)
Pain, Numbness	6 (14%)
Dry mouse, Oral discomfort	5 (12%)
Chest pain	5 (12%)
Excessive sweating	5 (12%)
Frequent urination	5 (12%)
Menstrual irregularities	3 (7%)
Globus sensation	2 (5%)
Difficulty swallowing	2 (5%)
Tremor	2 (5%)
Itching, Pain	2 (5%)
Difficulty speaking	1 (2%)
Residual urine sensation	1 (2%)
Erectile / Ejaculatory dysfunction	1 (2%)
Hypertension	0 (0%)
Difficulty urinating	0 (0%)

**Table 3:** Injection site, injection volume and adverse effects of epidural oxygen injection. Most procedures were performed via the sacral hiatus, and no serious treatment-related adverse events requiring hospitalization or resulting in permanent sequelae were observed. Data are presented as *n* (%). Patients could experience more than one adverse event.

Detail of Epidural Oxygen Injection	Value
**Injection site**	
Lumbar, *n* (%)	3 (7.1)
Sacral hiatus, *n* (%)	39 (92.9)
**Injection volume**	
40 mL, *n* (%)	2 (4.8)
50 mL, *n* (%)	2 (4.8)
60 mL, *n* (%)	14 (33.3)
70 mL, *n* (%)	18 (42.9)
80 mL, *n* (%)	6 (14.3)
**Adverse events**	
subcutaneous emphysema, *n* (%)	5 (11.9)
Dyspnea, *n* (%)	2 (4.8)
back pain, *n* (%)	1 (2.4)

**Table 4:** Changes in SF-36v2^®^ scores between pre- and post-treatment. Changes in all SF-36v2^®^ domains favored the post-treatment assessment, and all improvements remained statistically significant after Bonferroni correction for multiple comparisons. Data are presented as median (IQR). *p* values were calculated using the paired Wilcoxon signed-rank test and adjusted using the Bonferroni method.

SF-36v2^®^ Domain	Pre Median (IQR)	Post Median (IQR)	Post–Pre Median	*p* Value (Bonferroni-Corrected)	r
Physical functioning (PF)	70.0 (40.0–80.0)	75.0 (55.0–90.0)	5	0.0057	0.56
Social functioning (SF)	18.8 (3.1–34.4)	43.8 (21.9–75.0)	18.8	0.00034	0.7
Physical role limitation (RP)	41.0 (22.0–51.0)	61.0 (41.0–74.0)	12	0.023	0.51
Emotional role limitation (RE)	35.0 (20.0–45.0)	47.0 (35.0–54.5)	15	0.0011	0.61
Vitality (VT)	37.5 (12.5–50.0)	62.5 (25.0–75.0)	25	0.02	0.49
General health (GH)	45.0 (30.0–65.0)	60.0 (37.5–80.0)	10	0.0028	0.59
Mental health (MH)	18.8 (3.1–37.5)	43.8 (21.9–56.3)	18.8	0.00034	0.72
Body pain (BP)	41.7 (16.7–70.8)	58.3 (33.3–95.8)	8.3	0.0058	0.57

## Data Availability

The data presented in this study are available upon request from the corresponding author.
